# ManyFold: an efficient and flexible library for training and validating protein folding models

**DOI:** 10.1093/bioinformatics/btac773

**Published:** 2022-12-10

**Authors:** Amelia Villegas-Morcillo, Louis Robinson, Arthur Flajolet, Thomas D Barrett

**Affiliations:** InstaDeep, London W2 1AY, UK; Department of Signal Theory, Telematics and Communications, University of Granada, Granada 18071, Spain; InstaDeep, London W2 1AY, UK; InstaDeep, London W2 1AY, UK; InstaDeep, London W2 1AY, UK

## Abstract

**Summary:**

ManyFold is a flexible library for protein structure prediction with deep learning that (i) supports models that use both multiple sequence alignments (MSAs) and protein language model (pLM) embedding as inputs, (ii) allows inference of existing models (AlphaFold and OpenFold), (iii) is fully trainable, allowing for both fine-tuning and the training of new models from scratch and (iv) is written in Jax to support efficient batched operation in distributed settings. A proof-of-concept pLM-based model, pLMFold, is trained from scratch to obtain reasonable results with reduced computational overheads in comparison to AlphaFold.

**Availability and implementation:**

The source code for ManyFold, the validation dataset and a small sample of training data are available at https://github.com/instadeepai/manyfold.

**Supplementary information:**

Supplementary data are available at *Bioinformatics* online.

## 1 Introduction

The prediction of 3D protein structure from amino acid sequence is a challenging problem due to complex interactions between residues occurring in the space. Due to the close relationship between protein structure and biological function, methods that attempt to predict structures using only the amino acid sequence hold significant value, whilst still representing a significant practical challenge. In recent years, deep learning has emerged as the *de facto* paradigm of choice for so-called *ab initio* protein structure prediction, rapidly become the state-of-art approach.

In particular, AlphaFold v2 (Jumper *et al.*, 2021)—the most successful folding model to date—is able to generate plausible protein structures as a result of massive computation on large multiple sequence alignments (MSAs) and, optionally, templates of similar sequences with known structure. However, these inputs can be computationally expensive to generate, rely on searching large databases and can be of low quality for many rare proteins that lack known homologs.

An alternative is to take advantage of the vast amount of protein sequences contained in public databases to train protein language models (pLMs) (Elnaggar *et al.*, 2021; Rives *et al.*, 2021). Since the self-supervisory signal is the amino acid sequence itself, no structure information is needed to train pLMs. Moreover, the internal representations (or embeddings) learned by pLMs have proven successful in predicting the structural attributes of the protein. This is inspiring a new generation of folding models that replace the input MSAs with pre-trained pLM embeddings (Fang *et al.*, 2022; Lin *et al.*, 2022; Wu *et al.*, 2022).

The development of the next generation of protein folding models has been aided by the open-sourcing of AlphaFold (inference only), and, re-implementations such as OpenFold (inference and training code in PyTorch) (Ahdritz *et al.*, 2022). However, the immense complexity and computational cost associated with developing such models still present a significant bottleneck and challenge to the wider community. To support these efforts, in this work, we introduce ManyFold—a flexible protein folding library that can implement multiple models (both MSA-based and pLM-based), is fully trainable and highly efficient, supporting batched operations and distributed compute across platforms. As well as demonstrating our pipeline on existing AlphaFold and OpenFold models, we also train from scratch a proof-of-concept pLM-based model, pLMFold. Using ESM-1b (Rives *et al.*, 2021) embeddings and attention maps as input to a lightweight AlphaFold-inspired network, pLMFold obtains reasonable results while significantly reducing inference time.

## 2 Approach

ManyFold is implemented in Python and Jax (http://github.com/google/jax). It allows for distributed training and efficient batched validation on a variety of platforms, including graphical processing units (GPUs) and tensor processing units (TPUs). The library contains all necessary scripts for training full AlphaFold models from either randomly initialized parameters and optimizer state, a previously stored checkpoint or pre-trained model parameters (for model fine-tuning). In addition, it can perform batched inference on validation sets to obtain the predicted structures and confidence metrics. Similarly, validation and fine-tuning of OpenFold models are also allowed (see https://github.com/aqlaboratory/openfold on how to convert the model parameters into Jax).

**Fig. 1.**
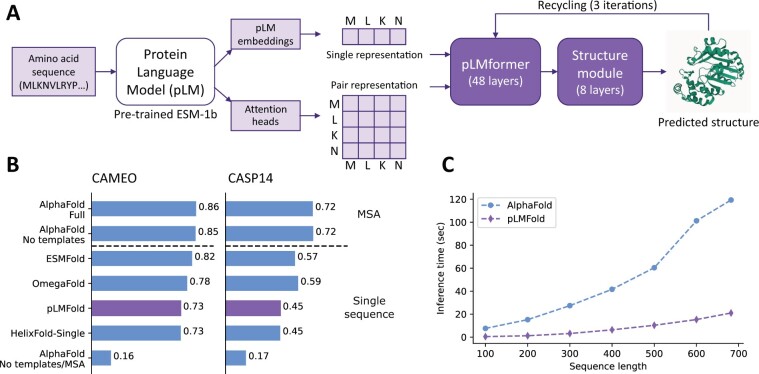
(**A**) The pLMFold model: a pre-trained pLM model first converts the input amino acid sequence into single and pair representations that are processed in the pLMformer module. The output is then fed into the structure module to generate the predicted protein structure. (**B**) Validation results measured as lDDT scores for our pLMFold model compared to other pLM-based models and AlphaFold (using either MSAs or single sequences as inputs). ‘Full’ refers to model_1_ptm and ‘No templates’ to model_5_ptm. (**C**) Inference times computed for pLMFold and AlphaFold over input sequences with different lengths. Inference times are averaged over five batches, each containing a single validation sample

Additionally, ManyFold includes a new model called pLMFold, which we introduce here (Fig. 1A). Unlike AlphaFold, which takes MSAs as inputs, the pLMFold model works on single protein sequences represented by pLM embeddings. While pLMFold can operate on any type of pLM embeddings, we use the ESM-1 family of models (Rives *et al.*, 2021) for this proof-of-concept (Jax implementations and weights are also provided). To process single sequences, we developed a more efficient version of the Evoformer, the pLMformer. Its inputs are (i) a single representation, which is a weighted average of the pLM embeddings over the different layers of the pLM and (ii) a pair representation that can be initialized to either the outer sum of the single representations or the averaged attention heads of the input pLM transformer. Then, the pLMformer applies multi-head attention over the single representation alone and reduces the model complexity by removing the triangle self-attention of AlphaFold which has computational cost scaling cubically with sequence length. This is in contrast to previous pLM-based prediction models [ESMFold (Lin *et al.*, 2022), OmegaFold (Wu *et al.*, 2022) and HelixFold-Single (Fang *et al.*, 2022)], where these computationally intensive operations are retained.

To validate ManyFold, we trained the pLMFold from scratch (using ESM-1b as pre-trained model and the original AlphaFold training data) on Google TPUs v2-128 (training details can be found in Supplementary material S1). Although pLMFold was trained using a subset of losses (structure loss, distogram loss and pLDDT loss), the tool supports training/fine-tuning on all losses defined for AlphaFold. The learning curves of pLMFold are provided in Supplementary Figure S1. We then validated this model along with pre-trained AlphaFold, OpenFold, ESMFold, OmegaFold and HelixFold-Single models using an NVIDIA A100 GPU. Note that, unlike these methods, our pLMFold model was not fine-tuned on larger crops and did not use predicted structures as distillation targets during training.

## 3 Results

The performance of our folding models is validated on CAMEO targets from March to May 2022 with less than 700 residues, and domains from CASP14 (see Supplementary material S2 for the full list of target ids). Figure 1B shows the lDDT scores (Mariani *et al.*, 2013) given by pLMFold and two AlphaFold models using MSAs or just the target sequence as input (labeled as ‘No MSA’). Extended results for both datasets—including other widely used metrics such as TM-score and GDT-TS—are provided in Supplementary Tables S1 and S2. Ultimately, the performance of AlphaFold drops significantly when only the target sequence is provided as input (similar results are obtained with the OpenFold parameters as detailed in Supplementary material S2). By contrast, the pLMFold achieves considerably better results with the same limited input information (recovering 81.4% of the performance on CAMEO of the full AlphaFold model using MSA and templates). Moreover, pLMFold outperforms the full AlphaFold model for ∼5% of the evaluated targets. We also note that pLMFold does not use the additional fine-tuning or self-distillation phases of the full AlphaFold pipeline.

Figure 1C plots the batched inference time as a function of sequence length, where the almost exponential scaling of AlphaFold inference time can be contrasted to the more linear scaling of pLMFold—culminating in a 6× speed for the longest targets. Indeed, this is a conservative estimate of the speed-up as the additional overheads of generating MSA and template features are not included for AlphaFold, whereas the calculation of ESM-1b embeddings is included for pLMFold.

## Supplementary Material

btac773_Supplementary_Data

## Data Availability

The data underlying this article are available at https://github.com/instadeepai/manyfold.
