# Supplementary material for: ManyFold: an efficient and flexible library for training and validating protein folding models
Source: Bioinformatics. 2022 Dec 10;39(1):btac773. doi: 10.1093/bioinformatics/btac773 (PMC9825755; doi:10.1093/bioinformatics/btac773)
Supplement: btac773_Supplementary_Data [file btac773_supplementary_data.pdf]

ManyFold: An efficient and flexible library for training and validating protein folding models
Supplementary Material

Amelia Villegas-Morcillo[1,2], Louis Robinson[1], Arthur Flajolet[1] and Thomas D. Barrett[1]

[1]InstaDeep, London, W2 1AY, UK and [2]Department of Signal Theory, Telematics and Communications, University of Granada, Granada, Spain
Contact: t.barrett@instadeep.com.

1 pLMFold hyperparameters and training losses

1.1 Hyperparameters

The main set of hyperparameters used to train the pLMFold model are listed below. For the complete list of hyperparameters, please refer to the configuration files in the GitHub repository.

General	Loss weights	Optimizer
Crop size pLM*: 1024<br>Crop size: 256<br>Batch size: 128<br>Recycling iterations: 3	Structure module: 1.0<br>Distogram: 0.3<br>pLDDT: 0.01	Initial learning rate: 0.001<br>Warm-up steps: 1000<br>LR decay: 0.95, after step: 50000<br>EWA decay: 0.999
**pLMformer module**	**Structure module**	
Number of blocks: 48<br>Single representation size: 256<br>Pair representation size: 128	Number of channels: 384<br>Number of layers: 8<br>Number of heads: 12	

*Note that the ESM-1b model was trained on crops up to 1024 residues.

1.2 Training losses

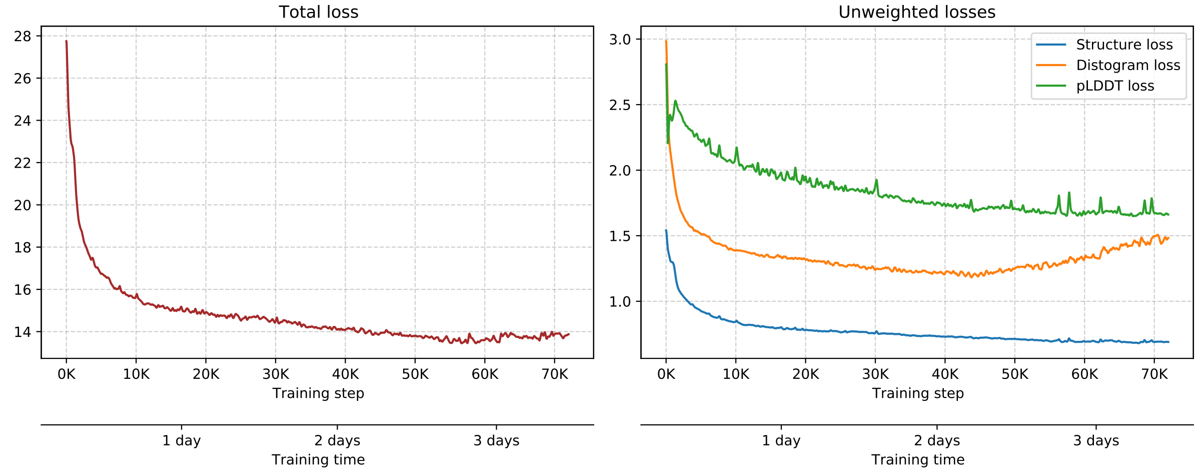

Figure S1: Training performance of the pLMFold model. For each training step, the total weighted loss value is shown on the left, while the values of the individual components (structure loss, distogram loss, and pLDDT loss) are shown on the right. Relative training time is also included, noting that convergence was reached in 3.5 days on the v2-128 TPUs. Also note that the total loss is computed as a sum weighted by uncropped sequence length (according to AlphaFold). All curves have been Gaussian-smoothed.

2 Training/validation sets and results

2.1 Training set

We use a procedure similar to the one put forth in [4] to collect the training set. Specifically, our training set is comprised of all structures of individual protein chains that can be extracted from entries in the Protein Data Bank (PDB) [2] with: (i) a release date before 2020-05-14 (2018-04-30 for the original Alphafold models [4]), (ii) a resolution less than 9 Å, and (iii) no single amino acid accounting for more than 80% of the sequence of the corresponding chain. This adds up to a total of $\sim$ 490k structures. The cutoff release date used to build the training set is prior to the CASP14 challenge in order to fairly compare with the performance of open-source models trained on data released before CASP14 took place, such as AlphaFold. Then, we use the same stochastic filters as in [4] to select which chains to train on during each pass over the full training set. Specifically: (i) protein chains are selected with probability $\frac{1}{512 \cdot C_{\text{size}}} \max(\min(N_{\text{res}}, 512), 256)$, where N_{res} is the number of amino acids in the chain and C_{size} is the size of the PDB cluster this protein chain falls into (clusters are derived using MMSeqs2 with a 40% sequence identity cutoff [7]).

2.2 Validation sets

CAMEO

As our primary validation set, we use the targets released as part of the CAMEO competition between March 2022 and May 2022, which include samples of the three levels of difficulty (easy, medium and hard). Note that the cutoff release date used to build the training set is prior to the release date of these targets.

List of targets in our CAMEO validation set:
`7EQH_A, 7ERO_A, 7ERN_C, 7FOA_A, 7F9H_A, 7MKU_A, 7OPB_D, 7POI_C, 7PRQ_B, 7PSG_C, 7SO5_H, 7TZE_C, 7TZG_D, 7X9E_A, 7Z5P_A, 7EQS_A, 7MLA_B, 7N29_B, 7NQD_B, 7OA7_A, 7Q4L_A, 7QAO_A, 7QAP_A, 7QRY_B, 7QS2_A, 7QS5_A, 7R5Z_B, 7R63_C, 7T4Z_A, 7TNI_C, 7VNX_A, 7YWG_B, 7CTX_B, 7EQE_A, 7ETS_B, 7F2Y_A, 7FEV_A, 7FJS_L, 7OB6_A, 7OD9_C, 7OVP_A, 7PB4_I, 7PXY_A, 7QIL_A, 7RPR_A, 7RPS_A, 7RQF_A, 7SCI_A, 7VGM_A, 7YXG_A, 7FOO_B, 7MHW_A, 7N3T_C, 7OSW_A, 7PC3_A, 7PC4_A, 7PC7_B, 7PC9_A, 7PNO_D, 7RI3_C, 7T7Y_A, 7U2R_A, 7V1K_A, 7X4E_A, 7ELF_C, 7EQB_A, 7MSK_A, 7OOB_A, 7Q4I_B, 7RAW_A, 7RPY_A, 7UGH_A, 7ULH_A, 7VNA_A, 7W5S_A, 7W5U_A, 7WME_A, 7XOD_A, 7Z79_B, 7EGT_B, 7EJG_C, 7FIW_B, 7M5W_A, 7O4O_A, 7POH_A, 7P3I_B, 7PW1_A, 7QBP_A, 7RO9_A, 7VU7_A, 7WNW_B, 7WWR_A, 7X8V_A, 7EFS_D, 7EHG_E, 7MYV_B, 7QO5_E, 7QDW_A, 7QSS_A, 7QSU_A, 7R74_B, 7S2R_B, 7TV9_C, 7V8E_B, 7VMC_B, 7VNO_A, 7W26_A, 7WWX_A, 7A67_A, 7A67_B, 7ERP_B, 7ESO_A, 7ETR_A, 7ETR_C, 7PC6_A, 7QBZ_A, 7RCZ_A, 7SGN_C, 7TCR_C, 7VWT_A, 7W1F_B, 7BOK_A, 7EBQ_A, 7ED1_A, 7ED6_A, 7F3A_A, 7PUJ_A, 7QBG_E, 7TBU_A, 7ACY_B, 7EAD_A, 7KO9_A, 7KOB_A, 7LXK_A, 7LXS_A, 7NOE_A, 7NUV_A, 7QDV_A, 7TXP_A, 7U5F_D, 7U5Y_A, 7V4S_A, 7WRK_A`

CASP14

As an additional validation set, we use the domain-level targets from the Free-Modeling (FM) and Template-Based Modeling hard (TBM-hard) categories of the CASP14 competition, only considering contiguous domains that are part of protein chains that have since been added to PDB. To generate the ground-truth structures for the domains, we align their sequences to the chains found in the corresponding PDB entries and discard domains that can be aligned with either no or more than one chain with 80% sequence identity.

List of targets in our CASP14 validation set:
`T1041-D1, T1039-D1, T1082-D1, T1049-D1, T1056-D1, T1030-D2, T1031-D1, T1035-D1, T1037-D1, T1053-D1, T1070-D3, T1099-D1, T1038-D2, T1042-D1, T1029-D1, T1038-D1, T1090-D1, T1054-D1, T1043-D1, T1055-D1, T1040-D1, T1078-D1, T1053-D2, T1032-D1, T1026-D1, T1074-D1, T1033-D1, T1030-D1, T1045s2-D1, T1065s2-D1, T1046s2-D1, T1047s1-D1, T1046s1-D1`

2.3 Extended results

We obtained validation results for our pLMFold model, and compared to several AlphaFold models (using either MSAs or single sequences as inputs). Tables S1 and S2 include the results for the CAMEO and

CASP14 datasets described above, respectively. The reported metrics are: lDDT (using all atoms or only C_α) [6], TM-score [10], GDT-TS, GDT-HA [9], and the average predicted LDDT (pLDDT) given by the models. For AlphaFold models, 'Full' refers to `model_1_ptm` and 'No templates' to `model_5_ptm`. We additionally validated both datasets on the full OpenFold model [1], as well as the first ESMFold [5], OmegaFold [8] and HelixFold-Single [3] models released. As can be seen, the targets in our CASP14 dataset are difficult to predict, resulting in an overall decrease in the performance of all models. This is particularly noticeable for our pLMFold model, but also for OmegaFold and HelixFold-Single. Nevertheless, these pLM-based models still achieve considerably better results than AlphaFold using single sequences as inputs.

Table S1: Validation results of the CAMEO dataset.

**Model**	**lDDT**	**lDDT-C_α**	**TM-score**	**GDT-TS**	**GDT-HA**	**pLDDT**
AlphaFold (Full)	0.8572	0.9157	0.8909	0.8714	0.7566	89.46
OpenFold (Full)	0.8546	0.9132	0.8892	0.8676	0.7498	89.86
AlphaFold (No templates)	0.8526	0.9103	0.8846	0.8634	0.7485	89.46
pLMFold	0.7313	0.7944	0.7675	0.7175	0.5828	81.36
ESMFold	0.8246	0.8830	0.8609	0.8305	0.7179	83.69
OmegaFold	0.7838	0.8455	0.8167	0.7831	0.6601	83.55
HelixFold-Single	0.7314	0.7917	0.7813	0.7351	0.5924	78.60
AlphaFold (No templates/MSA)	0.1591	0.1830	0.1353	0.1324	0.1007	39.66

Table S2: Validation results of the CASP14 dataset.

**Model**	**lDDT**	**lDDT-C_α**	**TM-score**	**GDT-TS**	**GDT-HA**	**pLDDT**
AlphaFold (Full)	0.7157	0.7816	0.7508	0.7284	0.6073	79.19
OpenFold (Full)	0.6815	0.7416	0.7031	0.6857	0.5677	78.73
AlphaFold (No templates)	0.7187	0.7826	0.7557	0.7347	0.6146	79.20
pLMFold	0.4497	0.5005	0.4313	0.3953	0.2844	61.56
ESMFold	0.5693	0.6271	0.5844	0.5565	0.4487	61.41
OmegaFold	0.5949	0.6513	0.6104	0.5841	0.4786	66.87
HelixFold-Single	0.4486	0.4805	0.4487	0.4146	0.2887	52.54
AlphaFold (No templates/MSA)	0.1677	0.1951	0.1524	0.1447	0.1103	44.96

References

[1] G. Ahdritz, N. Bouatta, S. Kadyan, Q. Xia, W. Gerecke, T. J. O'Donnell, D. Berenberg, I. Fisk, N. Zanichelli, B. Zhang, et al. OpenFold: Retraining AlphaFold2 yields new insights into its learning mechanisms and capacity for generalization. *bioRxiv*, 2022. doi: 10.1101/2022.11.20.517210.

[2] H. M. Berman, J. Westbrook, Z. Feng, G. Gilliland, T. N. Bhat, H. Weissig, I. N. Shindyalov, and P. E. Bourne. The Protein Data Bank. *Nucleic Acids Research*, 28(1):235–242, 2000.

[3] X. Fang, F. Wang, L. Liu, J. He, D. Lin, Y. Xiang, X. Zhang, H. Wu, H. Li, and L. Song. HelixFold-Single: MSA-free protein structure prediction by using protein language model as an alternative. *arXiv*, 2022. doi: 10.48550/ARXIV.2207.13921.

[4] J. Jumper, R. Evans, A. Pritzel, T. Green, M. Figurnov, O. Ronneberger, K. Tunyasuvunakool, R. Bates, A. Žídek, A. Potapenko, et al. Highly accurate protein structure prediction with AlphaFold. *Nature*, 596(7873):583–589, 2021.

[5] Z. Lin, H. Akin, R. Rao, B. Hie, Z. Zhu, W. Lu, A. d. Santos Costa, M. Fazel-Zarandi, T. Sercu, S. Candido, and A. Rives. Language models of protein sequences at the scale of evolution enable accurate structure prediction. *bioRxiv*, 2022. doi: 10.1101/2022.07.20.500902.

[6] V. Mariani, M. Biasini, A. Barbato, and T. Schwede. lDDT: a local superposition-free score for comparing protein structures and models using distance difference tests. *Bioinformatics*, 29(21):2722–2728, 2013.

[7] M. Steinegger and J. Söding. Clustering huge protein sequence sets in linear time. *Nature Communications*, 9(1):1–8, 2018.

[8] R. Wu, F. Ding, R. Wang, R. Shen, X. Zhang, S. Luo, C. Su, Z. Wu, Q. Xie, B. Berger, J. Ma, and J. Peng. High-resolution de novo structure prediction from primary sequence. *bioRxiv*, 2022. doi: 10.1101/2022.07.21.500999.

[9] A. Zemla. LGA: a method for finding 3D similarities in protein structures. *Nucleic Acids Research*, 31(13):3370–3374, 2003.

[10] Y. Zhang and J. Skolnick. Scoring function for automated assessment of protein structure template quality. *Proteins: Structure, Function, and Bioinformatics*, 57(4):702–710, 2004.
